# Limited Cutaneous Vasculitis Associated With Levamisole-Adulterated Cocaine

**DOI:** 10.4021/jocmr1003w

**Published:** 2012-09-12

**Authors:** Ralph Yachoui, Sharon L Kolasinski, Hala Eid

**Affiliations:** a Division of Rheumatology, Cooper Medical School of Rowan University, USA

**Keywords:** Cocaine, Vasculitis, Levimasole

## Abstract

Levamisole is among the many contaminants that have been detected in seized cocaine throughout North America and Europe. Little is known about the association between levamisole-adulterated cocaine and vasculitis. Herein we describe a case of limited cutaneous vasculitis manifested as retiform purpura and skin necrosis in a user of cocaine contaminated with levamisole.

## Introduction

Levamisole is a synthetic imidazothiazole derivative originally approved as an antihelminthic and subsequently used in the treatment of colon cancer, rheumatoid arthritis and pediatric nephrotic syndrome. Recent reports link the use of levamisole-laced cocaine to life-threatening agranulocytosis and to a distinctive form of necrotic purpura [1].

## Case Report

**Figure 1:**
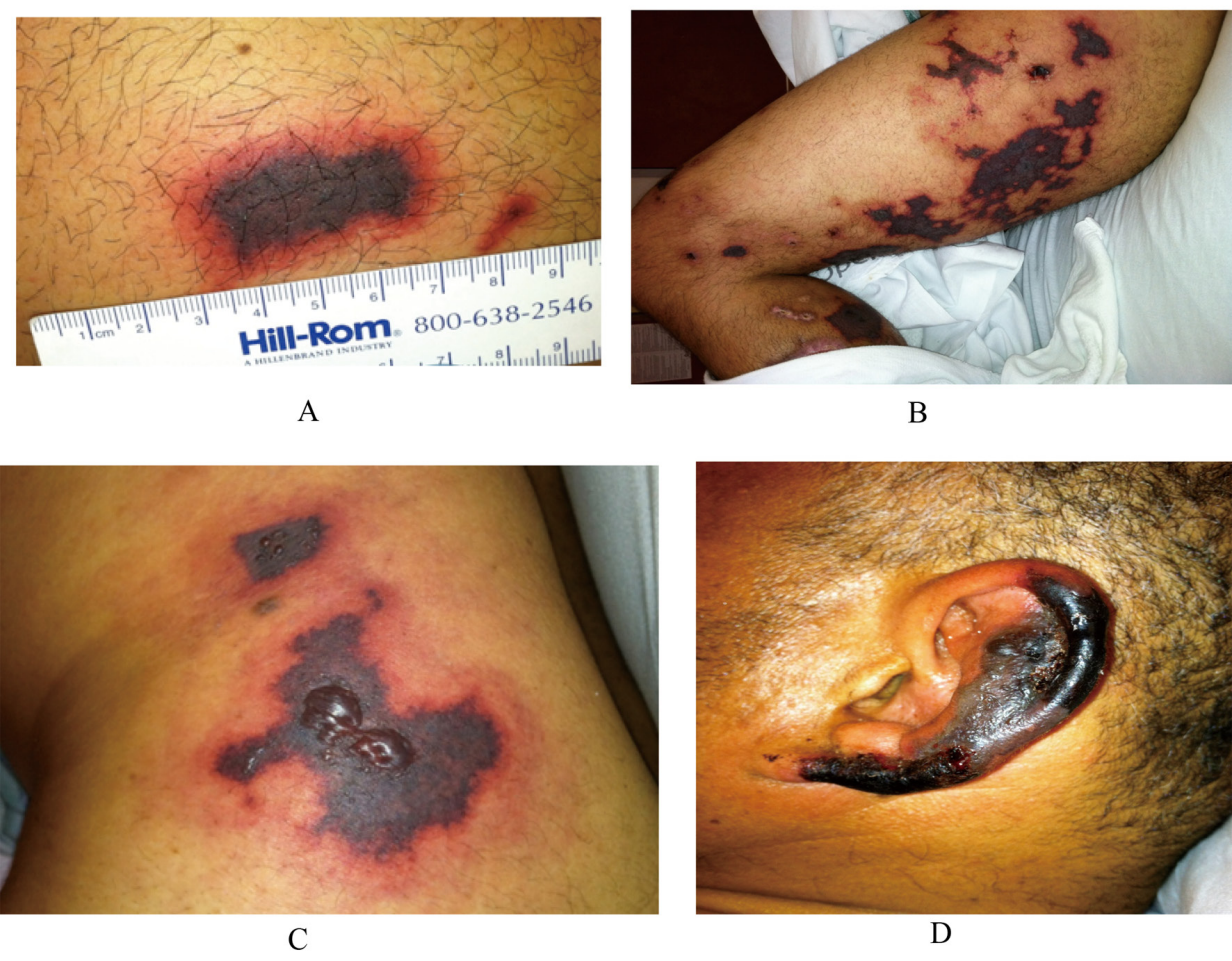
(A) Purpuric skin eruption - thigh. (B) Retiform purpura involving the entire lower extremity. (C) Necrotic retiform purpura with hemorrhagic bullae. (D) Ear necrosis.

A 43-year-old Hispanic male, previously healthy, was initially seen at our institution with 3-week history of a purpura affecting the arms and legs that progressed to necrosis and ulceration of the skin (Fig. 1A). He reported smoking ‘crack’ cocaine for 2 months. His review of systems was negative for evidence of internal organ involvement. Laboratory examination revealed elevated erythrocyte sedimentation rates and serum C-reactive protein levels. His complement C4 was low. He had a positive perinuclear antineutrophil cytoplasmic antibody (pANCA) serology with antibodies to myeloperoxidase (MPO) detected by enzyme-linked immunosorbent assays (ELISA).

He underwent a skin punch biopsy which demonstrated an acute neutrophilic necrotizing vasculitis involving the small and medium-vessel along with fibrinoid necrosis. He tested positive by urine toxicology for recent exposure to cocaine and to levamisole, as measured by gas chromatography and mass spectrometry. He was started on prednisone 60 mg daily. The skin lesions improved and he was discharged home on a quick taper dose of steroids.

Two months later, he was readmitted to our institution with worsening of his skin lesions. He stated he first noticed "dark spots" on his ears two weeks ago that resolved. One week prior to presentation he developed lesions on his ears, then on his lower extremities. The lesions were painful, changed from pink to purple to black, and also formed hemorrhagic bullae (Fig. 1B-D). At this time, he was neutropenic with the absolute neutrophil count at the lower limit of the normal range. His complement C4 was still low. A repeat serology revealed a positive lupus anticoagulant (LAC) test with positive pANCA and MPO. His urine drug screen (UDS) was positive for cocaine. Three days later, skin lesions remained stable and ear lesions improved with supportive treatment.

## Discussion

Levamisole has emerged as a prevalent adulterant of illicit cocaine in the US. A distinctive clinical syndrome including neutropenia and a purpuric skin eruption has been linked to levamisole-adulterated cocaine [1]. Neutropenia is a well-recognized toxicity of levamisole with the potential to be life-threatening. It has an overall incidence of 4.2% in all cocaine users [2]. The purpura is retiform, affecting the ears, face and extremities. It is different from the palpable purpura seen in ANCA-associated vasculitis and other small-vessel vasculitis [3].

The associated autoantibodies include ANA, LAC, IgM antibodies to cardiolipin, antibodies targeting multiple neutrophil antigens. This pattern of autoantibodies is similar to that seen in vasculitis induced by hydralazine, propylthiouracil, and other drugs [4]. The low complement in our case was not previously reported and may reflect an inherited deficiency which predispose to levamisole toxicity.

The predominant histopathology in the case reports and case series is a small-vessel thrombotic vasculopathy rather than vasculitis. Herein, we describe a vasculitis limited to the skin, without evidence of the involvement of other organ systems [1].

Discontinuation of the offending agent (cocaine and levamisole) is the mainstay of treatment. It is not known if systemic steroids are required for resolution. Rheumatologists should be aware of this distinctive form of cutaneous vasculitis associated with levamisole-adulterated cocaine.
